# The Influence of Heat Treatment on the Mechanical Properties of AlMn1Cu Aluminium Alloy with One-Sided AlSi7.5 Cladding Used in Heat Exchangers

**DOI:** 10.3390/ma18163915

**Published:** 2025-08-21

**Authors:** Martyna Zemlik, Beata Białobrzeska, Daniel Tokłowicz

**Affiliations:** 1 Department of Vehicle Engineering, Faculty of Mechanical Engineering, Wrocław University of Science and Technology, Wybrzeże Wyspiańskiego 27, 50-370 Wrocław, Poland; 2 Faculty of Mechanical Engineering, Wrocław University of Science and Technology, Wybrzeże Wyspiańskiego 27, 50-370 Wrocław, Poland

**Keywords:** 3xxx series aluminium alloys, static recrystallisation, mechanical properties, anisotropy

## Abstract

The aim of this study was to determine the influence of heat treatment parameters on the microstructure and mechanical properties of the AlMn1Cu (EN AW-3003) aluminium alloy with a one-sided cladding layer of AlSi7.5 alloy (EN AW-4343). The investigation was conducted within an annealing temperature range of 200 °C to 500 °C, analysing changes in hardness, mechanical strength, formability, and planar anisotropy. The results clearly indicate that within the temperature range of 300–340 °C, an intensive process of static recrystallisation occurs, leading to the restoration of a fine-grained and homogeneous microstructure. This is accompanied by a sharp reduction in hardness and yield strength, along with a significant increase in ductility and deep drawing capability. A notable reduction in the anisotropy of plastic properties was also observed, confirming effective homogenisation of the material’s microstructure. The findings unambiguously demonstrate that heat treatment within the range of 300–500 °C enables the formation of an isotropic microstructure with low hardness and high formability, rendering the material particularly suitable for shaping thin-walled components, including heat exchangers.

## 1. Introduction

A breakthrough in the development of the light metals industry came in 1886 with the invention of a method for aluminium production via electrolysis. This innovation enabled the industrial-scale production of aluminium, significantly reducing production costs and paving the way for its widespread application. By the end of the 19th century, aluminium had already found use in the manufacture of railway carriages (1894), car bodies (1899), and electrical transmission lines (1900). Since then, aluminium and its alloys have become among the most important structural materials in modern industry. Key physicochemical properties of aluminium include low density, high thermal and electrical conductivity, corrosion resistance, and excellent plastic workability. These characteristics make it ideally suited for use across numerous sectors such as automotive, aerospace, energy, construction, and the food industry [1,2]. As pure aluminium exhibits low strength, it is primarily used in the form of alloys in technical applications. These alloys, through carefully selected alloying elements, allow for modification of the microstructure and, consequently, the functional properties [3,4,5].

Based on the characteristics of phase equilibrium diagrams, aluminium alloys are divided into two main categories: casting alloys and wrought alloys, the latter of which includes alloys intended for further heat treatment. The 3xxx series, also known as Al-Mn alloys, belongs to this second category. The manganese content in these alloys typically does not exceed 1.5%, as limited solubility (max. 1.55 wt.% at the eutectic temperature) hampers eutectic formation. The addition of manganese enhances corrosion resistance and improves mechanical properties. Alloys from this series are used, among other applications, in the production of technical foils, kitchenware, roofing sheets, beverage cans, and components for heat exchangers [6,7].

Initially, heat exchangers were primarily manufactured using copper and its alloys, among which brass was the most valued. With technological advancement came the need for new solutions, which led to the adoption of alternative materials—in this case, aluminium. From an economic standpoint, aluminium is significantly less expensive than copper and its alloys, and its low mass contributes to the reduction of structural weight, which is of considerable importance in the automotive sector [8]. Consequently, aluminium alloy heat exchangers were introduced, produced using the method of expanded tubes and through brazing processes. A key material used for this purpose is aluminium alloy strip, which undergoes brazing during the manufacturing process.

One method of modifying the microstructure of 3xxx series alloys, and thus their mechanical properties, involves inducing microstructural transformations through the process of recrystallisation. Static recrystallisation (SRX) results in the elimination of internal stresses and enables the formation of a fine-grained microstructure characterised additionally by isotropy. In Al-Mn-Cu alloys, the SRX mechanism typically proceeds via heterogeneous nucleation of new grains on structural defects such as grain boundaries, shear bands, or areas of locally increased dislocation density. This process is strongly influenced by the degree of deformation, as well as the annealing time and temperature. The presence of finely dispersed secondary phase particles also plays a crucial role, as they may act as inhibitors of grain boundary migration, thereby stabilising the microstructure and limiting grain growth [9]. The evolution of microstructure under the influence of static recrystallisation in alloys of other series has been studied by, among others: Qian et al. [10], Zhang et al. [11], Huang et al. [12] and Gardner and Grimes [13]. In the case of 3xxx series aluminium alloys, aside from intensive grain refinement, an important factor influencing the microstructure and properties is the transformation of intermetallic phase particles occurring during annealing. The microstructure of these alloys contains primary particles of eutectic origin, including silicon, the cubic α-phase (with an approximate composition of Al_12–15_(Fe,Mn)_3_Si_1–2_) and the orthorhombic Al_6_(Fe,Mn) phase. The interrelationship among these constituents depends on both the silicon content and solidification conditions such as cooling rate. Notably, iron and manganese can partially substitute for each other in both phases, and the Fe:Mn ratio affects the crystalline form of the α-phase—higher manganese content favours a simple cubic structure, while greater iron content leads to a body-centred form. During annealing at temperatures around 350–400 °C, the Al_6_(Fe,Mn) phase transforms into the α-phase via a mechanism resembling a eutectoid reaction. This transformation occurs at the interface between the particle and the aluminium matrix, involving the diffusion of silicon, but also manganese, which influences the local Fe:Mn ratio. As the annealing temperature increases, fragmentation of eutectic colonies and their gradual spheroidisation occurs, with a simultaneous reduction in the number of particles and an increase in their average size. Concurrently with the transformation of primary particles, finely dispersed secondary particles—precipitates of the α-phase with a reduced Fe:Mn ratio—are formed within the aluminium matrix. This process primarily proceeds heterogeneously, initiated at dislocations, subgrain boundaries, and grain boundaries. The effectiveness of precipitation and the morphology of the dispersion depend on the alloy’s chemical composition, prior plastic and heat treatment, and the heating rate. High heating rates (above 20 K/s) can effectively suppress this process. The temperature range in which precipitation is most intense typically falls within 300–500 °C; however, at higher temperatures (above 540 °C), the smaller precipitates may redistribute into the matrix, increasing the manganese content in solid solution and promoting secondary growth of primary particles [14,15,16,17,18].

The AlMn1Cu alloy, which belongs to the 3xxx series, is used in the production of heat exchangers due to its high corrosion resistance and favourable mechanical properties [14]. However, its application in this role necessitates the use of a multilayer material, including clad aluminium sheets [16,17]. The cladding technique involves applying a thin layer of a different alloy—with distinct properties—onto the surface of the base alloy; in this case, the cladding alloy exhibits higher brazability. For materials intended for heat exchangers, a core–clad configuration is commonly used, where the core consists of the AlMn1Cu alloy and the cladding layer is made from a 4xxx series alloy, such as AlSi (4343), which is characterised by a lower melting point [19]. The cladding layer is most frequently applied using hot rolling, although other techniques such as cold rolling or casting are also possible. The melting temperature of the AlMn1Cu core is approximately 660 °C, while the cladding layer melts within the range of 560–600 °C. During the brazing process, this temperature is maintained for approximately four minutes. During this time, the cladding layer melts, allowing the components of the heat exchanger to bond [8]. The performance properties of clad aluminium materials depend largely on the parameters of the applied heat treatment.

The objective of this article is to determine the influence of annealing, conducted within the temperature range of 200–500 °C, on the mechanical and microstructural properties of AlMn1Cu alloy with a one-sided cladding layer of AlSi7.5 alloy. The evaluated parameters included the following: hardness, yield strength, tensile strength, percentage elongation at fracture, formability (assessed using the Erichsen test), and the degree of planar anisotropy. Although the mechanical behaviour of EN AW-3003 alloys during rolling and annealing has been addressed in previous studies, the present work differs in several important aspects that define its novelty and scope. The investigated material was not a monolithic EN AW-3003 alloy but a one-sided clad strip consisting of an EN AW-3003 core and an EN AW-4343 (AlSi7.5) cladding layer applied by industrial hot rolling. This configuration, common in automotive heat exchangers, has not been the subject of comprehensive studies assessing its mechanical response to post-rolling annealing. Furthermore, the starting strip underwent substantial cold deformation, with a 79% thickness reduction, a factor that has a profound impact on static recrystallisation behaviour and resulting mechanical properties but is often neglected in general studies of 3003 alloys.

In addition to hardness and tensile strength, the present work evaluates formability (Erichsen test) and planar anisotropy, providing a more complete characterisation of how annealing parameters affect the material’s suitability for complex forming operations in heat exchanger manufacturing. The results clearly identify 340 °C as the onset of full recrystallisation, yielding an isotropic, fine-grained microstructure that remains stable without abnormal grain growth up to 500 °C. This finding has direct technological significance, as it enables the design of annealing schedules that maximise formability and minimise anisotropy while avoiding detrimental grain coarsening.

Finally, this research was carried out in close collaboration with an industrial partner, driven by the practical need to establish safe and effective annealing parameters for clad AlMn1Cu/AlSi7.5 sheets. Such parameters were not available in the open literature for this specific product type, despite its widespread application and strong industrial demand.

## 2. Materials and Methods

**Figure 1:**
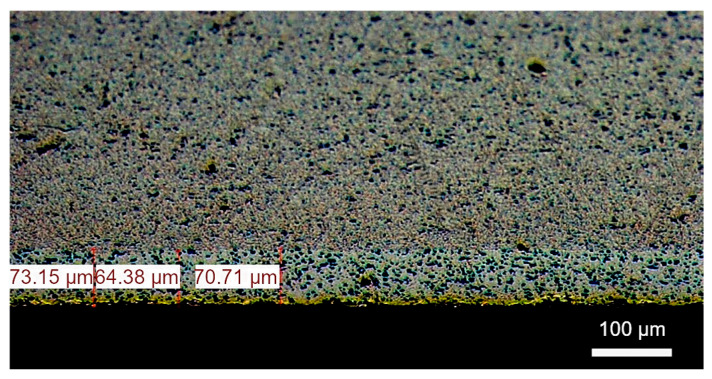
Analysed aluminium alloy AlMn1Cu with a one-sided cladding layer of AlSi7.5, with a thickness ranging from 64 to 73 µm.

The material used in the study was the AlMn1Cu (EN AW-3003) aluminium alloy, belonging to the group of non-heat-treatable alloys, characterised by an increased manganese content. The surface of the sample was clad on one side with a layer of AlSi7.5 alloy (EN AW-4343), with the aim of improving technological properties, particularly in terms of brazing and oxidation resistance. The chemical composition of the EN AW-3003 core alloy and the EN AW-4343 cladding alloy is presented in Table 1 and Table 2. The content of individual elements complies with the requirements of the EN 573-3:2019 standard [20]. The sheet thickness after plastic deformation was 1.2 mm, while the cladding thickness ranged from 64 to 73 µm, representing approximately 5.33–6.08% of the total strip thickness (Figure 1).

The heat treatment of the samples was carried out in a laboratory furnace manufactured by Nabertherm (Nabertherm GmbH, Lilienthal, Germany). The samples were heated together with the furnace from ambient temperature to the target annealing temperature, held for 4 h, and then air-cooled. Each sample was annealed separately at a specific temperature of 200, 220, 240, 260, 280, 300, 320, 340, 360, 380, 400, 420, 440, 460, 480, or 500 °C, corresponding to 20 °C increments.

Hardness measurements were carried out using a Zwick/Roell ZHU 250 hardness tester (ZwickRoell GmbH & Co. KG, Ulm, Germany). Material for testing was taken from both the core side and the cladding side. The Brinell method was applied in accordance with the ISO 6506-1:2014 standard [21].

The tensile test at ambient temperature was conducted in accordance with the current ISO 6892-1:2020 standard [22]. For this purpose, standard flat proportional tensile test specimens were prepared, with a thickness of 4 mm and a gauge length of *L*_0_ = 50 mm. Samples were cut in two rolling directions: 0° and 90°. The tests were carried out using a Zwick/Roell Z005 universal testing machine (ZwickRoell GmbH & Co. KG, Ulm, Germany) equipped with an extensometer with a gauge length of *L*_0_ = 50 mm. The tensile test was conducted at a constant strain rate, controlled based on the stress rate (Method B, as specified in ISO 6892-1:2020), until fracture occurred. The following fundamental mechanical properties of the material were determined: the conventional yield strength (*R_p_*_0.2_), ultimate tensile strength (*R_m_*), and percentage elongation after fracture (*A*).

The formability of the strip and sheet was tested in accordance with ISO 20482:2014 [23], using the classical Erichsen cupping test method. The purpose of this test was to evaluate the resistance of the material to plastic deformation during forming processes. This method is widely applied in studies concerning the formability of metallic materials, particularly sheet metals, and has been discussed in numerous research works addressing the influence of microstructure, alloying, or processing conditions on material behaviour during deformation [24,25,26,27,28,29]. During the test, the sample, clamped between a die and a blank holder, was subjected to loading by a hemispherical punch with a diameter of 20 mm. As the punch advanced, a dome-shaped indentation was formed on the surface of the sample. The test was terminated when the first crack appeared, penetrating the full thickness of the sheet. The measurable result was the so-called Erichsen number (IE), which refers to the punch penetration depth (expressed in millimetres) from initial contact with the sample to the moment of cracking. Prior to testing, the sample surface was lubricated with a graphite-based agent to reduce friction and ensure consistent loading conditions. Measurements were performed using a Erichsen 111 testing station (ERICHSEN GmbH & Co. KG, Hemer, Germany) designed for evaluating aluminium strips and sheets with thicknesses ranging from 0.1 to 2 mm. The device recorded the maximum punch depth at the moment of crack initiation, which allowed for the direct determination of the material’s formability.

The technological planar anisotropy index (*W*) was determined using the cupping test method in accordance with ISO 20482:2014, which is one of the recognised methods for evaluating the formability of the tested material. For this purpose, test cups were prepared using an Erichsen 143 type machine (ERICHSEN GmbH & Co. KG, Hemer, Germany). This device enabled both the punching of circular blanks and the forming of cups, which served as specimens for analysis. Measurement of the planar anisotropy index was performed using an Erichsen 126 type automatic device, specifically designed for this purpose. The device calculates the anisotropy index based on the measured cup heights, according to the following relationship (1):

(1) $$W=\frac{\sum h_{max}-\sum h_{min}}{\sum h_{max}}\times 100\%$$

where

h_max_—maximum cup height,
h_min_—minimum cup height.

In order to carry out microstructural observations, metallographic examinations of the samples were performed in two variants: on the cross-section (including the core layer) and on the rolled surface. Prior to observation, the samples underwent appropriate preparation, depending on the nature of the intended analysis. Samples intended for surface microstructure analysis were subjected to a two-stage electrochemical treatment. The first stage involved anodising for 2 min in a suitably selected electrolyte, aimed at enhancing the surface structure. This was followed by electropolishing for 1 min to obtain a homogeneous, smooth observational surface. For samples prepared for cross-sectional microstructure observations, a classical metallographic procedure was applied, including grinding with abrasive papers of increasing grit size, followed by mechanical polishing using a diamond suspension. Upon completion of mechanical preparation, all samples were thoroughly rinsed with distilled water and dried using a stream of compressed air. The final stage of preparation involved etching the sample surfaces with a specially formulated etchant, developed specifically for the purposes of this study to optimally contrast the microstructure of the tested alloy. Microstructural observations were conducted using a Nikon Epiphot 200 optical microscope (Nikon Corporation, Tokyo, Japan).

Light microscopy was deliberately selected as the primary microstructural characterisation technique in this study. This method offers sufficient resolution to evaluate the key features most relevant to the research objectives, including grain size, morphology, phase distribution, and the elimination of deformation-induced banding—the dominant microstructural factors influencing mechanical and formability properties within the examined annealing range. Although light microscopy cannot directly resolve nanoscale precipitates or dislocation structures, the microstructural changes most critical to the observed mechanical response are clearly identifiable using this approach. The primary objective was to determine the recrystallisation threshold and assess the stability of the fine-grained microstructure under conditions directly relevant to industrial annealing schedules. When combined with the mechanical testing, this methodology enabled the identification of dominant mechanisms, the determination of the recrystallisation threshold, and the evaluation of microstructural stability. While high-resolution techniques could provide additional insights into precipitation phenomena and defect structures, their application lay beyond the scope of the present work. Such advanced methods are planned for future studies to enhance understanding of the mechanisms—particularly those associated with precipitation—that contribute to the suppression of abnormal grain growth in this alloy.

## 3. Results

### 3.1. Relationship Between Mechanical Properties and Degree of Work Hardening

**Figure 2:**
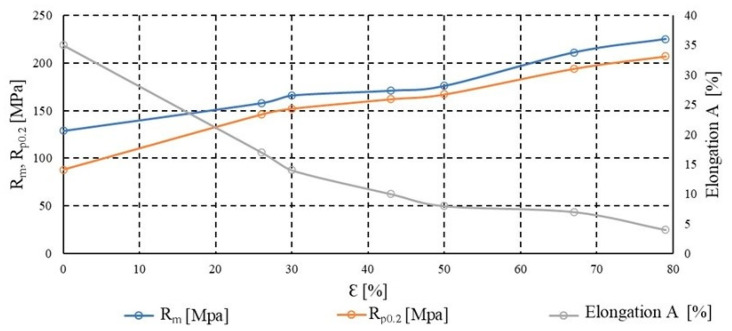
Dependence of the mechanical properties of AlMn1Cu with a one-sided cladding layer of AlSi7.5 on the degree of work hardening, longitudinal direction to the rolling direction.

**Figure 3:**
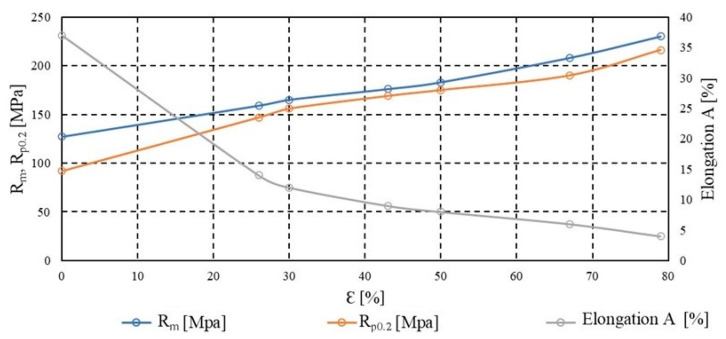
Dependence of the mechanical properties of AlMn1Cu with a one-sided cladding layer of AlSi7.5 on the degree of work hardening, transverse direction to the rolling direction.

Plastic deformation of metals, which is the permanent change in shape under stresses exceeding the yield strength, is associated with the movement of dislocations within the crystal lattice. As the degree of deformation increases—particularly in processes such as cold rolling—intense dislocation accumulation occurs within the material’s volume. These dislocations interact with one another, forming arrangements that obstruct further dislocation motion, resulting in an increased resistance of the material to deformation. This phenomenon is referred to as strain hardening, or work hardening, which manifests as an increase in strength parameters such as ultimate tensile strength (*R_m_*) and conventional yield strength (*R_p_*_0.2_), accompanied by a decrease in ductility. The rise in dislocation density primarily occurs along active slip planes and leads to the formation of substructures which, over time, may evolve into subgrain boundaries, initiating recovery and recrystallisation processes during subsequent heat treatment. Figure 2 and Figure 3 present the hardening curves of the material, illustrating changes in the basic mechanical properties–tensile strength, yield strength, and percentage elongation after fracture—as a function of deformation during cold rolling. The analysis was conducted on samples taken both along and across the rolling direction, which enabled the assessment of the influence of rolling orientation on mechanical properties. The initial thickness of the tested material was 6 mm. The rolling process was carried out progressively until a final thickness of 1.2 mm was achieved, corresponding to a 79% reduction in cross-sectional area. This reduction results in a true strain of approximately 1.609. In this condition, the material was subsequently subjected to annealing. With increasing deformation, a systematic rise in strength parameters was observed, accompanied by a decrease in elongation, which is characteristic of the strain hardening effect. The differences between the directions of testing also provided insight into the impact of structural anisotropy developed during the deformation process.

### 3.2. Influence of Recrystallisation Temperature on Mechanical and Microstructural Properties

#### 3.2.1. Hardness Measurements

The initial hardness of the tested material, measured on both the core and the clad side, was 59.8 HBW and 59.3 HBW, respectively. These values are characteristic of 3xxx series aluminium alloys that have previously undergone cold plastic deformation [30]. It should also be noted that due to the high repeatability of the results—and consequently a low standard deviation (at most 1 Brinell hardness unit or 2 MPa for parallel strength tests)—the authors refrained from conducting a statistical interpretation of the test data. The high initial hardness is directly associated with increased dislocation density, elongation and flattening of grains, and the presence of internal stresses resulting from work hardening. Such a microstructure contributes to elevated strength properties but simultaneously limits the material’s ductility and its suitability for further forming. With increasing annealing temperature, a systematic decrease in hardness was observed on both the core and clad sides. At 200 °C, the reduction in hardness was relatively minor–by only a few HBW units–suggesting that, at this stage, the dominant mechanism was the relaxation of internal stresses without significant microstructural changes. However, in the temperature range of 200–280 °C, a sharp drop in hardness occurred, from over 50 HBW to values close to 30 HBW. Such a sudden change indicates the onset of intense recrystallisation, during which new, finer, and more isotropic grains are formed.

Static recrystallisation, whose course can be inferred from this decline in hardness, is a process dependent on both temperature and soaking time. In the tested material, the critical point of this phenomenon appears to reach peak activity within the range of 260–300 °C. In this interval, the greatest differences in hardness relative to the initial state were recorded, which also correlates with a notable decrease in mechanical strength and an increase in formability, observed in parallel studies. Above 320 °C, hardness values stabilise and show no further significant fluctuations. The hardness of both the core and the cladding remains at approximately 30–32 HBW, which should be interpreted as the attainment of structural equilibrium. In this range, temperature no longer significantly influences microstructural transformations, the grains are fully recrystallised, and further increases in temperature do not initiate additional processes. The material thus enters a softened state, typical of annealed aluminium, with high plasticity. It is also worth noting the similar hardness values on both sides of the sample (the core and the cladding) which indicates effective thermal conductivity and a uniform course of annealing throughout the cross-section of the material. Despite the chemical composition differences between the two layers, their behaviour under heat treatment was highly comparable, which is particularly significant for further applications, such as brazing or the rolling of complex layered systems.

**Figure 4:**
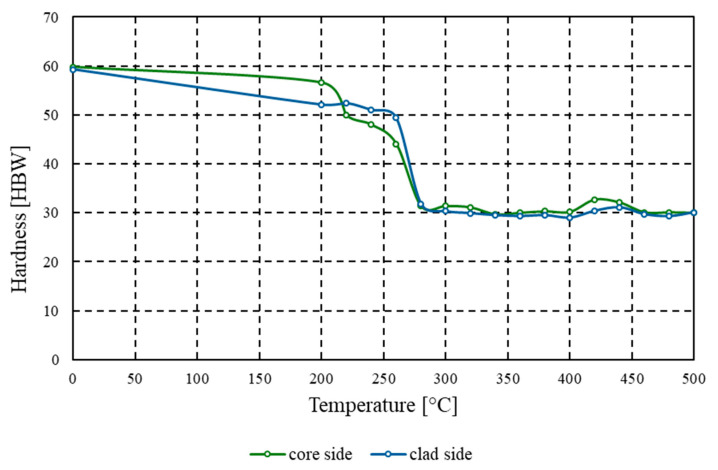
Graph of the dependence of hardness [HBW] on annealing temperature [°C] for the AlMn1Cu with a one-sided cladding layer of AlSi7.5, tested in both the longitudinal and transverse directions relative to the rolling direction.

From a practical perspective, the obtained hardness results indicate the existence of a clearly defined temperature range (300–500 °C) in which the material maintains a stable, low level of hardness that supports its plasticity. At the same time, they confirm that even relatively short soaking at temperatures around 260–300 °C can radically alter the mechanical properties of the material. In an industrial context, this knowledge is particularly valuable, as it allows for precise control over the functional properties of finished products, depending on specific technological requirements. Figure 4 presents the relationship between the average hardness of the AlMn1Cu alloy and the annealing temperature in the range of 200–500 °C, shown separately for measurements taken from the core side and the clad side. It should be emphasised that the reduction in hardness relative to the initial state amounted to nearly 50%.

#### 3.2.2. Static Tensile Test

**Figure 5:**
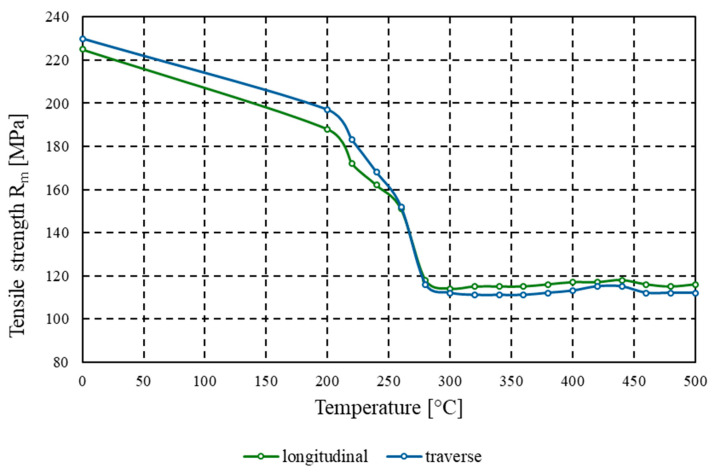
Graph of the dependence of tensile strength *R_m_* [MPa] on annealing temperature [°C] for the AlMn1Cu with a one-sided cladding layer of AlSi7.5, tested in both the longitudinal and transverse directions relative to the rolling direction.

Figure 5 presents the relationship between ultimate tensile strength (*R_m_*) and annealing temperature for samples taken in two directions of the plastically deformed sheet: longitudinal (marked in blue) and transverse (marked in green). According to the test results, the material in the cold-worked state exhibited a tensile strength of 225 MPa, while for samples tested transversely to the rolling direction, only a slight increase in this value was observed to 230 MPa, which indicates a low degree of anisotropy in the mechanical properties of the material. It should be noted that for the material annealed at 200 °C, tensile strength gradually decreased compared to the as-received state (to 188 MPa and 197 MPa for the longitudinal and transverse directions, respectively). A marked drop in tensile strength was observed only within the temperature range of 220–300 °C. For material annealed above 300 °C, the curve stabilised, and the tensile strength reached a plateau between 111 and 118 MPa for all samples subjected to heat treatment at various temperatures–up to the maximum annealing temperature of 500 °C.

**Figure 6:**
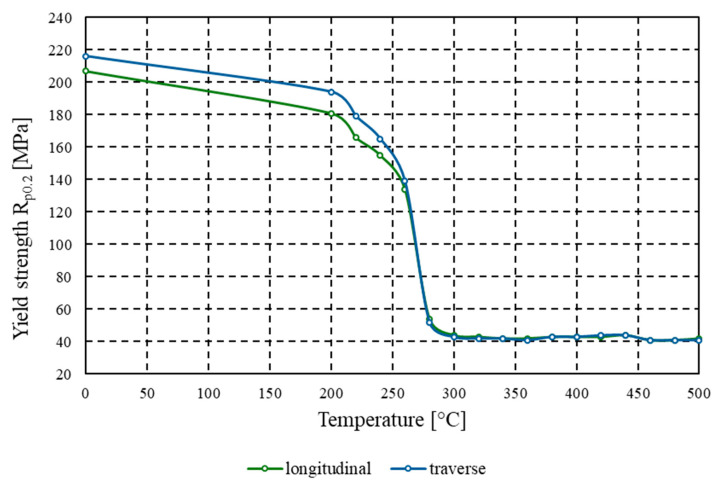
Graph of the dependence of conventional yield strength *R_p_*_0.2_ [MPa] on annealing temperature [°C] for the AlMn1Cu with a one-sided cladding layer of AlSi7.5, tested in both the longitudinal and transverse directions relative to the rolling direction.

The conventional yield strength (*R_p_*_0.2_) of the tested aluminium alloy prior to heat treatment was 207 MPa for material taken along the rolling direction, and 216 MPa for samples taken across the rolling direction (Figure 6). For material annealed at 200 °C, the yield strength decreased to 181 MPa (longitudinal direction) and 194 MPa (transverse direction). Annealing between 220 and 300 °C led to a sharp decline in yield strength for both longitudinal and transverse samples, with values stabilising in the range of 41–44 MPa. The curve plateaued, and these values did not change further with increasing annealing temperature. After annealing at 500 °C, an almost fourfold reduction in the material’s conventional yield strength was observed compared to the cold-worked state.

**Figure 7:**
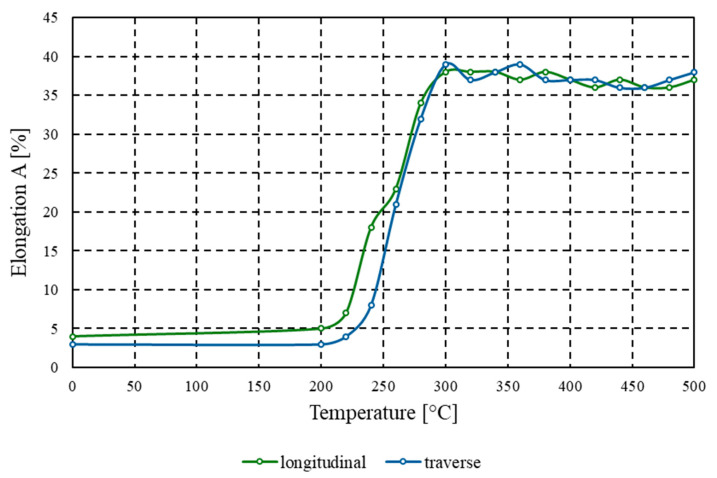
Graph of the dependence of percentage elongation after fracture *A* [%] on annealing temperature [°C] for the AlMn1Cu with a one-sided cladding layer of AlSi7.5, tested in both the longitudinal and transverse directions relative to the rolling direction.

In contrast to the behaviour of tensile strength and conventional yield strength, the percentage elongation after fracture *A* increased with rising annealing temperature (Figure 7). For samples tested in both the longitudinal and transverse directions relative to the rolling direction, the elongation prior to heat treatment was 4% and 3%, respectively. With increasing annealing temperature, the elongation value *A* rose nearly ninefold for the material annealed at 300 °C, reaching 39% and 38%, respectively. Further increases in the heat treatment temperature led to a stabilisation of the curve, with the measured values falling within the range of 36–39%.

#### 3.2.3. Formability Testing and Anisotropy Coefficient

**Figure 8:**
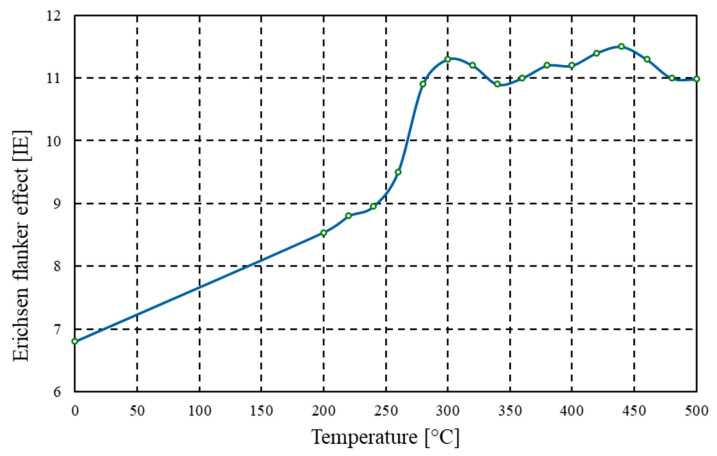
Graph of the dependence of Erichsen formability [IE] on annealing temperature [°C] for the AlMn1Cu with a one-sided cladding layer of AlSi7.5.

The results of the Erichsen formability test clearly indicate a significant influence of heat treatment temperature on the plastic deformability of the tested alloy (Figure 8). In the initial state, i.e., immediately after rolling, the punch penetration depth was 6.8 mm. This value is typical for strain-hardened materials, which exhibit increased hardness and residual internal stresses that limit the material’s ability to undergo local deformation under tensile loading conditions, as represented in the Erichsen test. For samples annealed at lower temperatures, i.e., within the range of 200–260 °C, the punch penetration depth ranged between 8.8 and 9.5 mm. These minor changes compared to the initial state suggest that full recrystallisation had not yet occurred within this temperature range. It may be assumed that only partial relaxation of internal stresses took place at this stage of the process, resulting in a slight improvement in deformability. It is also worth noting that the persistently high hardness of the material within this temperature range correlates with the limited increase in formability, which further confirms the strong dependence of deep drawability on the degree of microstructural softening achieved through annealing.

A significant improvement in the material’s formability was only recorded at annealing temperatures exceeding 300 °C. For samples soaked at 300–360 °C, the punch penetration depth began to increase markedly, reaching 11.0 mm after heat treatment at 360 °C. This abrupt increase in formability is attributed to the occurrence of complete recrystallisation processes within the material, leading to a reduction in dislocation density, restoration of a grain-structured microstructure, and a clear decrease in hardness and yield strength. Annealing within this temperature range also resulted in the homogenisation of the rolling texture, which further improved the ability of the material to deform isotropically under complex tensile stress conditions. The maximum formability value, amounting to 11.5 mm, was achieved after annealing at 440 °C. It should also be emphasised that further increases in annealing temperature up to 500 °C, did not result in significant changes in formability; the punch penetration depth remained at a stable, high level of approximately 11.0 mm. This indicates the completion of recrystallisation processes. The maintenance of elevated formability over a broad annealing temperature range (from 300 to 500 °C) is particularly advantageous from a technological standpoint. This property allows for increased flexibility in forming heat exchanger components, technical enclosures, tubular elements, or geometrically complex panels. Moreover, improved formability contributes to longer tool life in stamping operations and reduces the incidence of technological defects in finished products.

The planar anisotropy index represents an important parameter describing the degree of in-plane non-uniformity in the plastic properties of sheet material. It is a direct consequence of the crystallographic texture and the directional nature of the microstructure formed during rolling processes. For materials intended for deep drawing, it is essential that properties in the longitudinal, transverse, and diagonal directions relative to the rolling direction are as uniform as possible. High values of the *W* index indicate pronounced anisotropy, whereas low values suggest an isotropic structure that promotes uniform strain distribution and reduces the risk of defects such as “earing” or irregular edge deformations during forming operations.

**Figure 9:**
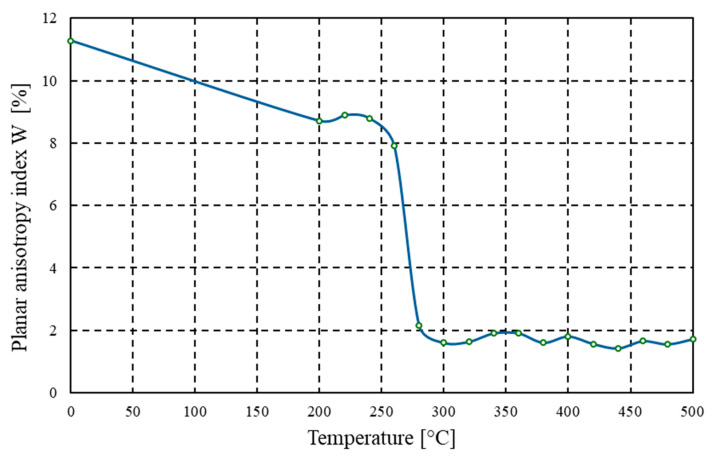
Graph of the dependence of the planar anisotropy index *W* [%] on annealing temperature [°C] for the AlMn1Cu with a one-sided cladding layer of AlSi7.5.

In the tested alloy, the planar anisotropy index (*W*) in the initial state was 11.27%, indicating a strong rolling texture and a variation in plastic properties depending on the sample orientation relative to the rolling direction (Figure 9). This level of anisotropy is typical for materials that have been heavily cold rolled, in which grains are significantly flattened and the crystallographic arrangement is aligned along a single preferred direction. As the annealing temperature increased, a clear downward trend in the W value was observed. Initially, at temperatures between 200–260 °C, the anisotropy value decreased only slightly, reaching levels of 8.7–7.9%. It may be assumed that within this temperature range, only partial stress relaxation and limited substructural transformations occurred, which is insufficient, however, for a significant reconfiguration of the texture. The recrystallisation process responsible for microstructural homogenisation and the elimination of directional orientation effects had not yet become fully active. It was only in the temperature range of 300–400 °C that a significant reduction in the anisotropy index was recorded–from the initial 11.27% to values below 2.0%–clearly indicating the occurrence of full recrystallisation and the formation of new grains with random crystallographic orientation. This process leads to a substantial reduction in the directionality of mechanical properties, which is particularly desirable in applications requiring deep drawing, especially in the production of thin-walled heat exchangers, technical packaging, or profiled components. The lowest *W* index values–below 1.71%–were recorded after annealing at temperatures between 420–500 °C, indicating the achievement of a nearly isotropic microstructure in the sheet plane. Such a material state enables more predictable forming of geometrically complex shapes, reduces the risk of local cracking and distortion, and minimises the need for adjustments to forming tools.

#### 3.2.4. Microscopic Examination

**Figure 10:**
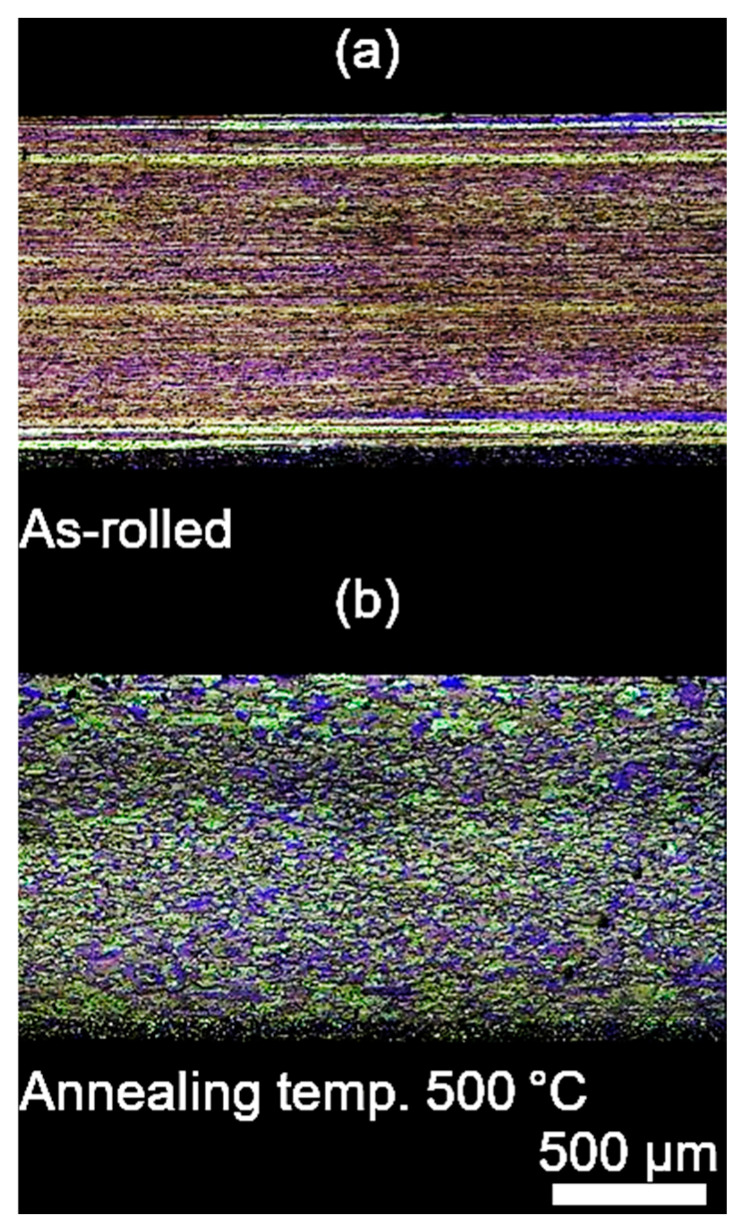
Microstructure on the cross-section of cold-rolled AlMn1Cu alloy with a one-sided cladding layer of AlSi7.5: (**a**) followed by four-hour isothermal annealing at 500 °C, (**b**). Etched condition, LM.

**Figure 11:**
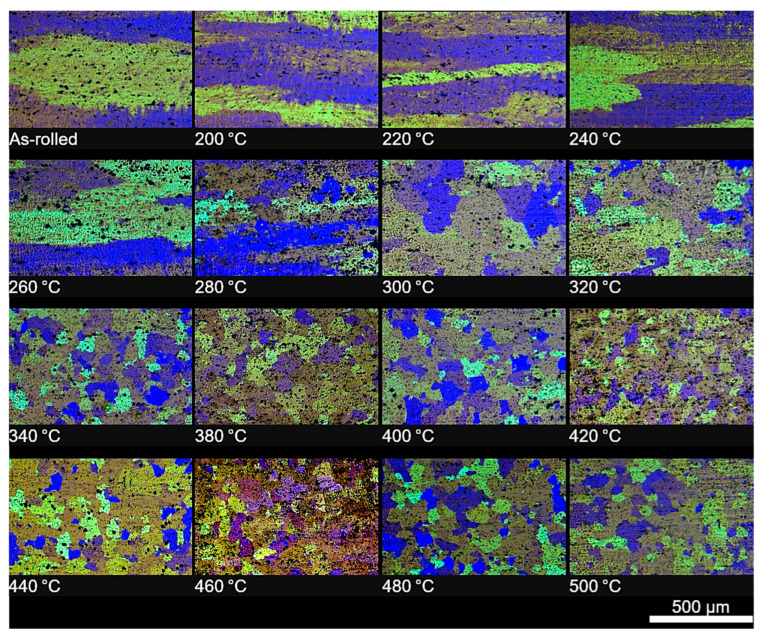
Microstructures of cold-rolled AlMn1Cu alloy, followed by four-hour isothermal annealing. Etched condition, LM.

To assess the influence of annealing temperature on the microstructure of the tested alloy, microscopic observations were carried out on the cross-section and surface of the samples after heat treatment. For each analysed annealing temperature, microphotographic documentation was prepared. Additionally, for comparative purposes, the microstructure of the sample in the initial state (prior to heat treatment) was recorded (Figure 10 and Figure 11). In the initial state, the microstructure displays pronounced banding, characteristic of both the cross-section and the surface of the material, which results from prior plastic deformation. Within the annealing temperature range from 200 °C to approximately 280 °C, no significant changes in microstructural morphology were observed–the banded structure remained stable, suggesting that recrystallisation had not yet commenced in this temperature range. The first distinct signs of recrystallisation initiation were observed only after annealing at 300 °C. New grain nuclei began to appear in the microstructure, primarily located at the boundaries between deformed grains. The microstructure of the material became uniform and fine-grained. As the annealing temperature increased, the recrystallisation process intensified. Analysis of microstructures obtained after annealing at 340 °C indicated that under these conditions the material underwent complete recrystallisation. The observed microstructure is characterised by the total disappearance of the original banded structure and the presence of uniform, fine, and evenly distributed grains. This clearly indicates that 340 °C represents the threshold temperature for full recrystallisation in the tested material.

It should be noted, however, that as in other studies on low-alloy cold-rolled sheet, the course of recrystallisation may vary depending on the degree of deformation. In the case of samples with a low degree of strain (ε < 0.9), there is a risk that even after annealing at high temperatures (up to 500 °C), only the surface layer of the material will be transformed, through grain boundary growth rather than the true recrystallisation mechanism. In such cases, the bulk of the sample may retain characteristics of a cast or deformed structure, as confirmed by both cross-sectional observations and local hardness measurements. This phenomenon was not observed in the present study, which suggests that the level of deformation was sufficient to activate full recrystallisation both at the surface and within the bulk of the material. Nonetheless, this factor should be taken into account when planning industrial annealing of thin aluminium strip (particularly in cases of low strain) where full microstructural uniformity may not be achieved through heat treatment alone [17].

## 4. Discussion

**Figure 12:**
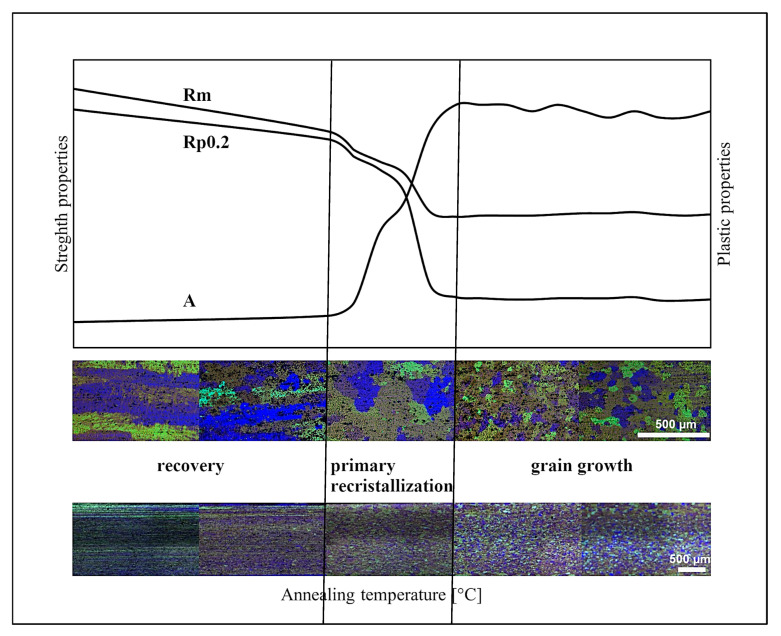
Schematic of changes in the properties and microstructure of AlMn1Cu alloy as a function of recrystallisation annealing temperature.

The results of the conducted study clearly indicate that the behaviour of the AlMn1Cu alloy with a one-sided cladding layer of AlSi7.5 in response to annealing falls within the typical range of phenomena described for high-formability 3xxx series aluminium alloys. The observed microstructural, mechanical, and technological changes are consistent with the mechanisms of static recrystallisation (SRX) and microstructural transformations involving finely dispersed particles, as described by Pokova et al. [14,15], Dehmas et al. [16] and Liu and Radhakrishnan [18]. Within the temperature range of 300–340 °C, an intensive recrystallisation process was identified, leading to a radical restructuring of the microstructure, from deformed bands to a fine-grained structure with low anisotropy (Figure 12). In the lower range of annealing temperatures, thickening of the deformed bands was observed, resulting from stress relaxation and partial recovery, without any significant restructuring of grain boundaries. As the temperature increased, these bands began to take on a more regular, equilibrium-like grain morphology, indicating an early phase of microstructural evolution. Nucleation of new, undeformed grains (unambiguously marking the onset of primary recrystallisation) was only observed after annealing above 300 °C.

In comparison with in situ studies (e.g., Dehmas et al. [16]), which enable real-time tracking of microstructural evolution during heating, it should be noted that the ex situ methodology applied in the present study provides a comprehensive snapshot of the post-process state but does not capture the kinetics of transformations in real time. Nevertheless, the observed changes (disappearance of bands, formation of large, irregular transitional grains, followed by the stabilisation of a fine-grained microstructure) are consistent with the theoretical stages of static recrystallisation, which are also confirmed by observations in related materials. In samples heated beyond 440 °C, no significant grain growth was observed, which distinguishes the investigated alloy from typical materials prone to uncontrolled grain coarsening post-recrystallisation. The resulting microstructure, following the completion of recrystallisation, is characterised by uniform grains and the absence of signs of abnormal grain growth, indicating effective suppression of grain boundary migration by secondary phase particles present in the matrix, as described by the research group of Pokova [14] and Nandihalli [31]. In the present study, no notable grain growth was detected above 340 °C, which may suggest effective microstructural stabilisation by the finely dispersed particles discussed in the aforementioned studies. The fully recrystallised microstructure obtained at 340 °C is characterised by uniform grains and a substantial reduction in hardness, which corresponds to the observations of Birol [17], who also identified a similar temperature range as typical for the initiation of static recrystallisation in supersaturated Al-Mn alloys. This observation is further supported by the corresponding reduction in yield strength and tensile strength. Another significant aspect of the results is the observed decrease in the planar anisotropy coefficient (*W*) from 11.3% to values below 2% after annealing at temperatures above 340 °C. This phenomenon confirms the effective homogenisation of crystallographic orientation, validating the efficacy of recrystallisation in eliminating the rolling texture. This is similar to findings reported by Pokova et al. [14,15].

Furthermore, in light of the study by Rusza et al. [32], which demonstrated that severe plastic deformation techniques can result in ultrafine-grained microstructures in AlMn1Cu alloy, the annealing effects obtained in the present work within the 340–440 °C range may be regarded as an industrially viable alternative–enabling the production of material with comparable properties using standard heat treatment methods. Although the resulting microstructure is not ultrafine-grained, as is typical of dynamic recrystallisation, it nonetheless offers high ductility and a low level of anisotropy, which may be sufficient for many industrial applications. In high stacking fault energy aluminium alloys such as Al-Mn, it is worth comparing, within the context of microstructural evolution in 3xxx series alloys, the mechanism of static recrystallisation (dominant in the investigated material) with the mechanism of continuous dynamic recrystallisation, typically observed under severe plastic deformation at elevated temperatures. As described by Kaibyshev and Malopheyev [33], this process proceeds without a distinct nucleation stage for new grains. Instead, there is a gradual increase in misorientation angles of subgrain boundaries, which transform into high-angle boundaries. As a result, new grains gradually emerge within the structure, ultimately leading to the formation of an ultrafine-grained microstructure. In dynamic recrystallisation, the microstructure evolves through the progressive transformation of low-angle boundaries into high-angle boundaries, ultimately giving rise to ultrafine grains. A key factor promoting continuous dynamic recrystallisation is the presence of deformation bands, geometrically necessary boundaries, and a suitable dispersion of secondary phase particles, which stabilise the grain boundary network and hinder migration [33]. Recent studies have shown that the addition of small amounts of alloying elements such as copper may influence the kinetics of development of both low- and high-angle grain boundaries. At the same time, the development of modern severe plastic deformation techniques, such as ECAP (Equal Channel Angular Pressing), makes it possible to effectively generate ultrafine-grained microstructures in AlMn1Cu alloys as well, significantly increasing their hardness without a substantial loss in ductility [32]. In the case of the investigated alloy, this was not the dominant mechanism of microstructural transformation, as the material was subjected to conventional heat treatment following cold rolling. A characteristic feature of static recrystallisation (SRX) is the appearance of grain nuclei in areas with locally elevated dislocation density, followed by their rapid growth until the deformed microstructure is entirely replaced. The results obtained in the present study, particularly the sudden drop in hardness and the change in microstructural morphology within a narrow temperature range (300–340 °C), confirm the operation of the SRX mechanism with a clearly defined initiation and completion threshold. It is also noteworthy that both the core and cladding sides exhibited a similar response to annealing, which may be attributed to good thermal conductivity and the relatively small thickness difference between the layers. Similar conclusions were drawn by Lee et al. [34], who, in their study of Al3003/Al4004 bimetallic materials, observed comparable kinetics of hardness and microstructural changes in both layers.

## 5. Conclusions

The aim of this study was to determine the influence of heat treatment parameters on the microstructure and mechanical properties of the AlMn1Cu aluminium alloy with a one-sided cladding layer of AlSi7.5. Particularly notable changes were observed within the temperature range of 300–340 °C, during which static recrystallisation was initiated and progressed. This process was accompanied by a distinct change in microstructural morphology, the banded microstructure was completely replaced by a uniform, fine-grained structure with equiaxed grains. Based on the analysis of the results, it was determined that full recrystallisation of the tested alloy occurred at 340 °C. Further increases in temperature did not result in significant changes in microstructure or mechanical properties, which allows this point to be defined as the threshold for optimal heat treatment conditions for the analysed material.

The experimental findings for AlMn1Cu with a one-sided AlSi7.5 cladding layer lead to the following conclusions:

(1) Tensile strength tested both in the longitudinal and transverse directions relative to the rolling direction systematically decreased with increasing soaking temperature from 225 MPa to 114 MPa (longitudinal) and from 230 MPa to 111 MPa (transverse). The conventional yield strength (*R_p_*_0.2_) for both longitudinal and transverse samples decreased from initial values of 207 MPa (longitudinal) and 216 MPa (transverse) to 41 MPa.
(2) Above the recrystallisation temperature (340 °C), further changes were minor, with tensile strength remaining within the range of 115–118 MPa and yield strength (*R_p_*_0.2_) within the range of 41–44 MPa.
(3) For the core layer, hardness dropped from 59.8 HBW to 29.7 HBW at 340 °C, and for the clad side from 59.3 HBW to 29.0 HBW at 400 °C. Above the recrystallisation temperature, the hardness stabilised within the range of 29.0–31.1 HBW.
(4) Annealing at 340 °C produced a nearly tenfold increase in percentage elongation compared to the as-rolled state. Whereas, material formability, as assessed by the Erichsen test, reached a maximum value of 11.5 mm after annealing at 440 °C. Above the recrystallisation temperature, further improvements were minimal, indicating that the plastic properties had stabilised.
(5) Heat treatment results in the elimination of the banded structure and the development of a uniform fine-grained microstructure. Contrary to some reports in the literature, soaking above the temperature of full recrystallisation does not induce secondary recrystallisation, which could otherwise lead to selective growth of certain grains and the development of a coarse-grained structure.

This study provides new insight into the annealing response of a heavily cold-worked EN AW-3003 aluminium alloy AlMn1Cu with a one-sided cladding layer of AlSi7.5 used in industrial production of automotive heat exchangers. The results demonstrate that prior deformation strongly affects static recrystallisation kinetics, enabling the identification of a precise full recrystallisation threshold at 340 °C. Above this temperature, the alloy exhibits a fine-grained, isotropic microstructure, stable mechanical properties, and enhanced formability without abnormal grain growth up to 500 °C. By combining mechanical testing, formability assessment, planar anisotropy measurements, and microstructural characterisation, the work delivers practical guidelines for optimising annealing schedules in industrial applications. While the present characterisation, based on optical metallography and mechanical testing, provided clear correlations between process parameters, grain morphology, and mechanical performance, higher-resolution techniques would offer further insight into precipitation phenomena and defect structures. Such advanced methods are planned for future studies to elucidate in greater detail the mechanisms—particularly those related to precipitation—that contribute to the suppression of abnormal grain growth, thereby supporting further optimisation of annealing schedules for industrial applications.

## Figures and Tables

**Table 1:** Chemical composition of the core and content of individual elements in accordance with EN-573:3 2019.

Chemical Composition of the Core in Accordance with EN-573:3 2019										
EN-573:3 2019	Si	Fe	Cu	Mn	Mg	Cr	Ni	Zn	Ti	Rest
	0.6	0.7	0.05–0.20	1.0–1.5	-	-	-	0.10	-	0.15
Chemical composition of the core obtained in the casting process										
EN AW-3003	Si	Fe	Cu	Mn	Mg	Cr	Ni	Zn	Ti	-
	0.475	0.546	0.101	1.253	0.006	0.014	0.0065	0.051	0.030	-

**Table 2:** Chemical composition of the cladding and content of individual elements in accordance with EN-573:3 2019.

Chemical Composition of the Cladding in Accordance with EN-573:3 2019										
EN-573:3 2019	Si	Fe	Cu	Mn	Mg	Cr	Ni	Zn	Ti	Rest
	6.8–8.2	0.8	0.25	0.10	-	-	-	0.20	-	0.15
Chemical composition of the cladding obtained in the casting process										
EN AW-4343	Si	Fe	Cu	Mn	Mg	Cr	Ni	Zn	Ti	-
	7.631	0.228	0.012	0.010	0.010	0.002	0.004	0.015	0.027	-

## Data Availability

The original contributions presented in this study are included in the article. Further inquiries can be directed to the corresponding authors.

## References

[1] Jasiński R., Chruścielski G. (2024). Possibilities to Modify the Properties of the AW7075 Aluminum Alloy for the Automotive Industry. Combust. Engines.

[2] Olaleye K., Roik T., Kurzawa A., Gavrysh O., Pyka D., Bocian M., Jamroziak K. (2022). Tribosynthesis of Friction Films and Their Influence on the Functional Properties of Copper-Based Antifriction Composites for Printing Machines. Mater. Sci.-Pol..

[3] Russell A.S. (1997). Aluminum.

[4] Schatzberg E. (2003). Symbolic Culture and Technological Change: The Cultural History of Aluminum as an Industrial Material. Enterp. Soc..

[5] Roik T.A., Gavrysh O.A., Vitsiuk Y.Y. (2019). The Functional Properties Acquired by Antifriction Composites Produced from Silumin Grinding Waste. Powder Metall. Met. Ceram..

[6] Lachowicz M.M., Lachowicz M.B., Gertruda A. (2022). Assessment of the Possibility of Galvanic Corrosion in Aluminum Microchannel Heat Exchangers. Crystals.

[7] Peta K., Siwak P., Grochalski K. (2017). Research on Mechanical Properties of Aluminum Alloys Used in Automotive Industry. Mater. Eng..

[8] Gutkowski K., Butrymowicz D. (2016). Refrigeration and Air Conditioning.

[9] Li X.S., Wu L.Z., Chen J., Zhang H.B. (2010). Static Softening Characteristics and Static Recrystallization Kinetics of Aluminum Alloy A6082 after Hot Deformation. J. Shanghai Jiaotong Univ. Sci..

[10] Qian X., Parson N., Chen X.G. (2020). Effects of Mn Content on Recrystallization Resistance of AA6082 Aluminum Alloys during Post-Deformation Annealing. J. Mater. Sci. Technol..

[11] Zhang T., Lu S.-H., Zhang J.-B., Li Z.-F., Chen P., Gong H., Wu Y.-X. (2017). Modeling of the Static Recrystallization for 7055 Aluminum Alloy by Cellular Automaton. Model. Simul. Mater. Sci. Eng..

[12] Huang C., Jia X., Zhang Z. (2018). Modeling and Simulation of the Static Recrystallization of 5754 Aluminium Alloy by Cellular Automaton. Metals.

[13] Gardner K.J., Grimes R. (1979). Recrystallization during Hot Deformation of Aluminium Alloys. Met. Sci..

[14] Poková M., Cieslar M., Lacaze J. (2011). Enhanced AW3003 Aluminum Alloys for Heat Exchangers. WDS.

[15] Poková M., Cieslar M., Slámová M. (2009). The Influence of Dispersoidson the Recrystallization of Aluminium Alloys. Int. J. Mater. Res..

[16] Dehmas M., Archambault P., Serriere M., Aeby-Gautier E., Gandin C.-A. (2002). In-Situ Precipitation in the Al-Mn-Fe-Si Alloy during Homogeneisation Treatment. Alum. Dusseld. Then Isernhag..

[17] Birol Y. (2008). Recrystallization of a Supersaturated Al–Mn Alloy. Scr. Mater..

[18] Liu W.C., Radhakrishnan B. (2010). Recrystallization Behavior of a Supersaturated Al–Mn Alloy. Mater. Lett..

[19] Mirski Z., Pabian J., Wojdat T. (2021). Dissolution and Erosion Phenomena in the Brazing of Aluminum Heat Exchangers. Arch. Metall. Mater..

[20] (2019). Aluminium and Aluminium Alloys—Chemical Composition and Form of Wrought Products—Part 3: Chemical Composition and Form of Products.

[21] (2014). Metallic Materials—Brinell Hardness Test—Part 1: Test Method.

[22] (2020). Metallic Materials—Tensile Testing—Part 1: Method of Test at Room Temperature.

[23] (2014). Metallic Materials—Sheet and Strip—Erichsen Cupping Test.

[24] He H., Yang T., Ren Y., Peng Y., Xue S., Zheng L. (2022). Experimental Investigation on the Formability of Al-Mg Alloy 5052 Sheet by Tensile and Cupping Test. Materials.

[25] Singh M., Choubey A.K., Sasikumar C. (2017). Formability Analysis of Aluminium Alloy by Erichsen Cupping Test Method. Mater. Today Proc..

[26] Vijaya A., Sanapala S., Arvind K.J., Darshan V. (2022). Vision based formability testing of sheet metal using portable Erichsen cupping tester. Mater. Today Proc..

[27] Singh J., Kim M.S., Lee S.E., Kim E.Y., Kang J.H., Park J.H., Kim J.J., Choi S.H. (2018). Heterogeneity in deformation and twinning behaviors through the thickness direction in E-form Mg alloy sheets during an Erichsen test. Mater. Sci. Eng. A.

[28] Hamada A.S., Kisko A., Khosravifard A., Hassan M.A., Karjalainen L.P., Porter D. (2018). Ductility and formability of three high-Mn TWIP steels in quasi-static and high-speed tensile and Erichsen tests. Mater. Sci. Eng. A.

[29] Sorce F.S., Ngo S., Lowe C., Taylor A.C. (2019). Quantification of coating surface strains in Erichsen cupping tests. J. Mater. Sci..

[30] Jankauskas V., Žunda A., Katinas A., Tučkutė S. (2025). Wear Study of Bulk Cargo Vehicle Body Materials Used to Transport Dolomite. Coatings.

[31] Nandihalli N., Guo Q., Gorsse S., Khan A.U., Mori T., Kleinke H. (2016). Thermoelectric Properties of Ni0.05Mo3Sb5.4Te1.6 with Embedded SiC and Al_2_O_3_ Nanoparticles. Eur. J. Inorg. Chem..

[32] Rusz S., Cizek L., Salajka M., Tylsar S., Kedron J., Michenka V., Donic T., Hadasik E., Klos M. (2014). Ultrafine Grain Refinement of AlMn1Cu and AZ 31 Alloys by SPD Process. Arch. Metall. Mater..

[33] Kaibyshev R., Malopheyev S.S. (2014). Mechanisms of Dynamic Recrystallization in Aluminum Alloys. Mater. Sci. Forum.

[34] Lee J.K., Lee S.P., Lee J.S., Lee S., Jo I., Bae D.S. (2020). Change of Microstructure and Hardness of Duo-Casted Al3003/Al4004 Clad Material during Extrusion Process. Metals.

